# Pile Damage Detection Using Machine Learning with the Multipoint Traveling Wave Decomposition Method

**DOI:** 10.3390/s23198308

**Published:** 2023-10-08

**Authors:** Juntao Wu, M. Hesham El Naggar, Kuihua Wang

**Affiliations:** 1 College of Civil Engineering and Architecture, Zhejiang University, Hangzhou 310058, China; 2 Geotechnical Research Centre, Western University, London, ON N6A 5B9, Canada

**Keywords:** pile integrity test, traveling wave decomposition, data-driven modeling, machine learning, damage characterization

## Abstract

The in-hole multipoint traveling wave decomposition (MPTWD) method is developed for detecting and characterizing the damage of cast in situ reinforced concrete (RC) piles. Compared with the results of MPTWD, the results of the in-hole MPTWD reconstruction technique are found ideal for evaluating the lower-part pile integrity and are further utilized to establish a data-driven machine-learning framework to detect and quantify the degree of damage. Considering the relatively small number of field test samples of the in-hole MPTWD method at this stage, an analytical solution is employed to generate sufficient samples to verify the feasibility and optimize the performance of the machine learning modeling framework. Two types of features extracted by the distributed sampling and statistical and signal processing techniques are applied to three machine-learning classifiers, i.e., logistic regression (LR), extreme gradient boosting (XGBoost) and multilayer perceptron (MLP). The performance of the data-driven machine-learning framework is then evaluated through a specific case study. The results demonstrate that all three classifiers perform better when employing the statistical and signal processing techniques, and the total of 24 extracted features are sufficient for the machine-learning algorithms.

## 1. Introduction

Very large capacity reinforced concrete (RC) piles are increasingly applied in modern engineering structures as an efficient and reliable foundation solution. This means fewer piles are used to support the structure, which dictates special attention to the quality control of their construction. Over the past few decades, the pile integrity test (PIT) [1,2,3,4] and cross-hole logging (CSL) [5,6,7] methods were developed for evaluating the construction quality of cast in situ RC piles. However, there still exist some common problems. The pile integrity test cannot be applied to extremely long piles, piles with severe defects near the pile top or when the pile heads have not been removed yet. Meanwhile, the cross-hole logging is not available when the reserved tubes are partially blocked. Additionally, the potential defect can only be located but cannot be characterized by both methods.

The parallel seismic (PS) method [8,9,10,11,12,13,14,15] has received more attention in recent years because it can resolve some of the above problems. However, compared with the PIT and CSL methods, the PS method requires a borehole parallel to the pile to conduct the test, which introduces more complicated preparations and higher costs. More recently, the multipoint traveling wave decomposition (MPTWD) method was proposed by Wu et al. [16] to evaluate the integrity of an extended pile shaft that supports a superstructure during its service life [17,18,19,20]. The MPTWD method can eliminate the influence of superstructure vibration while maintaining almost all the mechanical information below the transducers and, thus, can highlight the reflections caused by potential defects as well as the pile toe. The equipment required for the method is slightly different than those used for conventional PIT equipment. The advantages of the MPTWD method are summarized as follows:

(1) It eliminates the dynamic vibration effect of the superstructure above the transducers while maintaining almost all the mechanical information of the pile’s lower part;
(2) It implements the well-developed pile dynamic theory, which is the same as the conventional PIT and lateral PIT, and has the same capabilities, such as including damage detection and length prediction;
(3) It utilizes standard and uniform fictitious excitations, whose duration period can be as short as possible to distinguish the reflections and can be manually controlled;
(4) It employs equipment compatible with the conventional PIT or lateral PIT method [21,22].

In this study, the MPTWD method is further applied to the detection and characterization of the potential damage of cast in situ reinforced concrete (RC) piles and the lower-part pile integrity test (LPPIT) for extremely long piles. It may also be applied to situations involving piles with multiple defects, a pile head that has not been removed yet and the reserved sonic logging tubes that are partially blocked during the construction period. The in-hole MPTWD result reconstruction technique is ideal for evaluating the integrity of the pile’s lower part as it can detect any defect below the transducers as well as the pile toe. Additionally, the construction quality of RC piles is still evaluated by human experience [23] because there is no existing basis for its quality classification. Therefore, the in-hole MPTWD result is further applied to establish a data-driven machine-learning framework to characterize the quantity/degree of the potential damage. 

Since the number of field test samples of the in-hole MPTWD method is relatively small at this stage, an analytical solution is employed to generate sufficient samples to verify the feasibility and optimize the performance of the data-driven machine-learning framework. Two specific techniques for feature extraction are introduced to reduce the number of features that are applied to machine-learning algorithms: (1) distributed sampling, which is close to the conventional way of visual detection observed by the operators; and (2) the statistical and signal processing techniques, incorporating the Fourier transform (FT) and wavelet transform (WT). The wavelet transform is a mathematical operation where a scaled and shifted predefined mother wavelet is convoluted over a specified signal to find their similarity and is often utilized to detect signal mutations. The selection of the optimal mother wavelet that is commonly used for structural damage detection [24,25,26,27], e.g., Symlets 2 (sym2), Daubechies 5 (db5) and Daubechies 6 (db6), for the given task is also investigated in this study. The extracted features are applied to three machine-learning classifiers, i.e., logistic regression (LR), extreme gradient boosting (XGBoost) and multilayer perceptron (MLP). 

Sufficient samples generated by the analytical solution are utilized to tune the hyper-parameters of the machine-learning algorithms, including the C value (inverse of regularization strength) and regularization techniques of the LR classifier [28,29], the number of trees and max depth of the XGBoost classifier [30,31] and the Alpha value (penalty term) and hidden layer structures of the MLP classifier [32,33,34]. The performance of the machine-learning framework is then evaluated through the analysis of a case study.

## 2. In-Hole Multipoint Traveling Wave Decomposition (MTPWD) Method

### 2.1. Operation Steps

The in-hole MPTWD method involves three major steps: the installation of vibration transducers, test excitation and signal acquisition and the interpretation of collected signals. The details of these steps are provided below.

**Figure 1:**
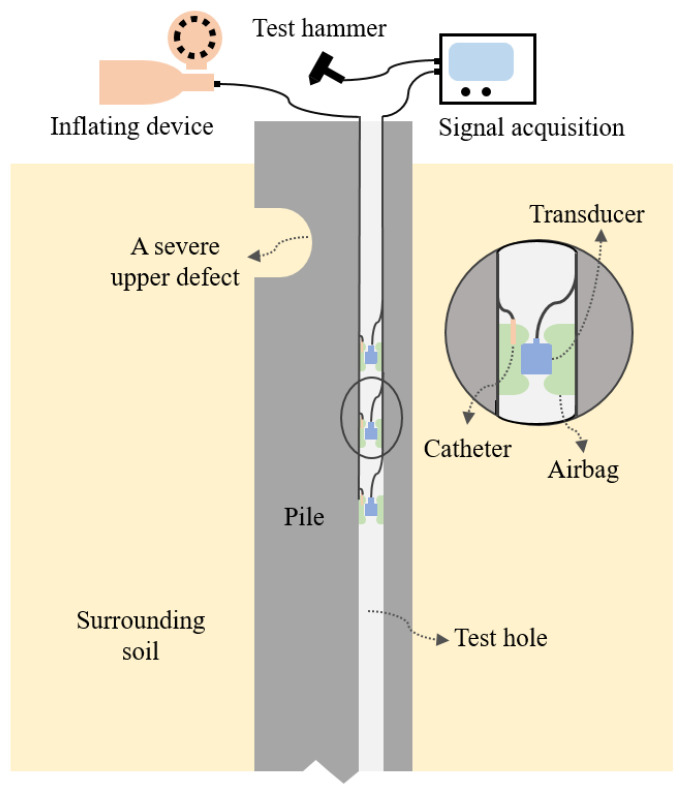
Schematic of the in-hole MPTWD method (transducers are located by inflating device).

(1) Pile preparation and transducer layout: As shown in Figure 1, multiple (at least three) equally-spaced acceleration/velocity transducers are located in a cased test hole (which can be replaced by the sonic logging tube if it exists) inside the test pile by clamping or inflating the device, and the fixtures are designed to be as lightweight and stiff as possible. The spacing between adjacent transducers should be larger than the product of the one-dimensional elastic wave velocity of the RC pile and the sampling time step but limited to 1.0 m to weaken the dissipation effect [16]. To better locate and characterize defects or to determine the pile toe, the transducers should be installed more than 1.0–2.0 m above the potential defect/pile toe. Specifically, for an extremely long pile, the transducers should be installed at the lower part to collect the pile toe reflection; for a pile with an enlarged pile head or severe upper defects, the transducers should be installed below the suspected defect location and the lower-part pile integrity test (LPPIT) should be applied.
(2) Test excitation and signal acquisition: A vertical impulse is imposed at the pile top to conduct the in-hole MPTWD method, and the transducers collect vertical velocity responses simultaneously. The selection of test impulse parameters and the high-frequency (HF) disturbance have an insignificant effect on the final MPTWD result [16]; therefore, a relatively low-frequency test impulse can be used (i.e., an incident wave with a longer duration, which corresponds to a deeper influence depth) to detect the lower-part pile integrity.
(3) Interpretation of in-hole MPTWD results: The dynamic responses of transducers are acquired and utilized to separate the downward and upward waves. By employing discrete Fourier transform (DFT) or fast Fourier transform (FFT), the pseudo-frequency response (PFR) function is defined by dividing the upward wave by the downward wave in the frequency domain. An idealized semi-sine fictitious excitation is then utilized to reconstruct the in-hole MPTWD results, which are close to conventional PIT results. Similar to the PIT method, it should also be noted that the accuracy of the in-hole MPTWD result is also restricted by the wavelength of the one-dimensional elastic wave. This method can eliminate the dynamic effect of the upper part of the pile foundation and, hence, distinguish the reflection caused by the first defect below (or by the pile toe for an intact pile). For a pile with multiple defects, the pile defects can be observed one by one from top to toe by lowering the transducers and repeating the test. The data processing procedures (including traveling wave decomposition and in-hole MPTWD result reconstruction) are demonstrated in the following sections.

### 2.2. Data Processing

(1) Traveling wave decomposition

The velocity time history collected by each transducer can be formulated as the sum of the downward and upward waves, i.e.,

(1) $$v\left(z,t\right)=\xi \left(z-C_{p}t\right)+\eta \left(z+C_{p}t\right),$$

where *ξ*(·) and *η*(·) are the downward and upward traveling waveforms, respectively; *C_p_* denotes the one-dimensional elastic wave velocity of the RC pile.

The effects of pile damping and soil resistance are negligible as the spacing between adjacent transducers is relatively small; therefore, it is reasonable to assume the traveling waves are not dissipated while passing through the transducers. Thus, for every three neighboring transducers, they satisfy the relation (*m* = −1, 0, 1):

(2) $$v\left(m,n\right)=\xi \left(m,n\right)+\eta \left(m,n\right)=\xi \left(z_{0}+m\Delta z,t+n\Delta t\right)+\eta \left(z_{0}+m\Delta z,t+n\Delta t\right)$$

where *z*_0_ denotes the depth of the middle transducer; Δ*z* is the spacing between adjacent transducers; Δ*t* = Δ*z*/*C_p_*; *n* is a nonnegative integer because of the causality of the linear time-invariant (LTI) system.

According to the traveling wave theory, the downward and upward waves yield the equations:

(3) $$\left\{\begin{array}{l} \xi \left(m,n\right)=\xi \left(m-1,n-1\right)\\ \eta \left(m,n\right)=\eta \left(m+1,n-1\right) \end{array}\right.,n\geq 1.$$

By comparing the velocities of the lower (*z*= *z*_0_ + Δ*z*) and upper (*z*= *z*_0_ − Δ*z*) transducers at *t* = *t* + Δ*t* with the velocity of the middle transducer (*z* = *z*_0_), we have:

(4) $$\left\{\begin{array}{l} v\left(1,1\right)-v\left(0,0\right)=\eta \left(1,1\right)-\eta \left(0,0\right)=\eta \left(0,2\right)-\eta \left(0,0\right)\\ v\left(-1,1\right)-v\left(0,0\right)=\xi \left(-1,1\right)-\xi \left(0,0\right)=\xi \left(0,2\right)-\xi \left(0,0\right) \end{array}\right..$$

Considering that *v*(0, 0), *v*(1, 1) and *v*(−1, 1) are known data collected by neighboring transducers, the downward and upward waves passing through the middle transducer can then be decomposed and reconstructed as demonstrated by Wu et al. [16].

(2) In-hole MPTWD result reconstruction

The separated downward and upward waveforms are utilized to develop the pseudo-frequency response (PFR) function, i.e.,

(5) $$H_{v}=\frac{\bar{\eta }\left(0,0\right)}{\bar{\xi }\left(0,0\right)}+1,$$

where *ξ*(0, 0) and *η*(0, 0) are the Fourier transform (FT) of *ξ*(0, 0) and *η*(0, 0), respectively.

In Equation (5), a unity is added to reproduce the downward (incident) wave, which makes the PFR function closer to the conventional frequency response (FR) function. The PFR contains almost all the mechanical properties information of the pile below the transducers.

An idealized semi-sine fictitious excitation, *f*, is subsequently induced to reconstruct the MPTWD results, i.e.,

(6a) $$v=\mathrm{IFT}\left[H_{v}\cdot \bar{f}\right]$$

(6b) $$\bar{f}=F\left[\sin\left(\theta_{f}t\cdot H\left(\frac{\pi }{\theta_{f}}-t\right)\right)\right],$$

where *v* denotes the dimensionless velocity response at the middle transducer due to the fictitious excitation; *f* is the Fourier transform of fictitious excitation whose amplitude is one; *θ_f_* is the angular frequency of the fictitious excitation; *H*(·) denotes the Heaviside step function. 

It should be noted that the time variable *t* can be chosen by the user, which overcomes the restriction of the instrument sampling frequency and facilitates standardization. The obtained results would be similar to the results derived from a free-top PIT or lateral PIT [21] applied to a pile foundation without a superstructure on its head.

**Figure 2:**
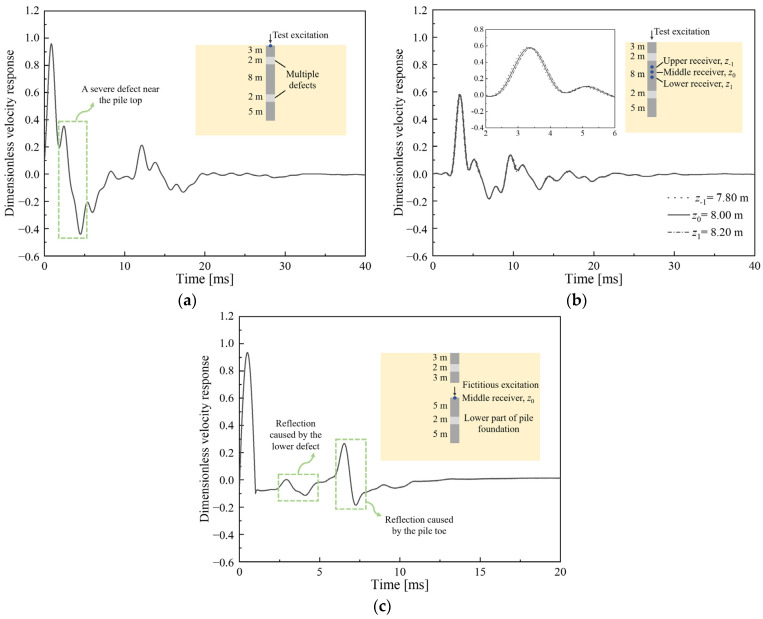
Data processing of the in-hole MPTWD method: (**a**) traditional PIT result; (**b**) time history velocities collected by three evenly-spaced transducers inside the pile foundation; and (**c**) the in-hole MPTWD result (data simulated by analytical solutions as solved in Section 4.1).

Figure 2a–c display, respectively, the traditional PIT results, time history velocities collected by three evenly-spaced transducers inside the pile foundation and the in-hole MPTWD results after the data processing procedure. It is hard to distinguish from the collected signals (Figure 2a), the pile toe reflection and the reflections caused by multiple defects because there exists a severe defect near the pile top. However, by introducing the traveling wave decomposition and in-hole MPTWD result reconstruction techniques, the in-hole MPTWD results (Figure 2c) derived from the collected signals (Figure 2b) are ideal for evaluating the lower-part pile integrity and distinguishing the reflections of the first defect below the transducers and the pile toe.

## 3. Data-Driven Modeling Framework

As demonstrated in Equations (6a) and (6b), the fictitious excitation, as well as the time interval of the MPTWD results, can be determined manually by the user. This means the MPTWD result can be preprocessed (standardized) and is, therefore, ideal for developing the necessary data for the following data-driven modeling framework. On the contrary, the conventional PIT or lateral PIT results have certain limitations in that regard: (1) the sampling time step is not always the same for different cases and equipment; (2) the duration of impulse excitation induced by a test hammer is inconsistent for each impact; and (3) the instrument drift and the disturbance of the environment can mislead the observation of the operator. Therefore, the interpretation of the PIT or lateral PIT results basically relies on the operator (i.e., subjective), and it is difficult or even impractical to characterize the quantity/degree of the pile damage through naked eyes. Thus, taking advantage of the developed method, and to overcome the limitations of the conventional PIT or lateral PIT method, the in-hole MPTWD result is further applied to establish a data-driven machine-learning framework and to characterize the quantity/degree of the potential damage as well.

**Figure 3:**
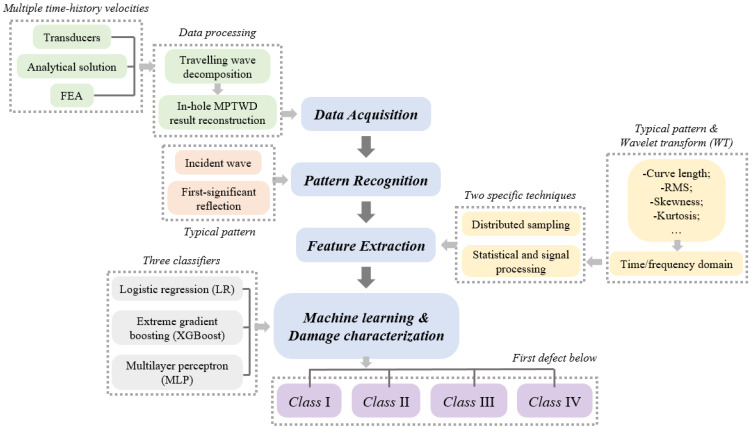
Flowchart of data-driven modeling framework.

A pile damage characterization framework is developed based on data-driven machine-learning analysis of the in-hole MPTWD results. The framework incorporates four major modules, as shown schematically in Figure 3 and described below:

(1) Data acquisition: The in-hole MPTWD results, i.e., the velocity time history of the lower-part pile foundation subjected to the idealized semi-sine fictitious excitation, are inputted as the raw data for the modeling framework. Because the developed method can suppress the effect of random uncertainty in the field test (e.g., impulse duration, high-frequency noise, zero/temperature drift, etc.), Wu et al. [16] reported that the MPTWD results derived from the field transducers are in good agreement with that obtained from data simulated by analytical solutions or finite element analysis (FEA). Given that the number of field test samples of the in-hole MPTWD method is relatively small at this stage, a large sample set is generated employing the analytical solution established in this study, and the feasibility of the data-driven modeling framework is demonstrated using this data set.
(2) Pattern recognition: The typical pattern of the lower-part pile integrity can be recognized by the waveform between the incident wave (i.e., the idealized semi-sine fictitious excitation) and the first significant reflection caused by the pile toe, or by a severe pile defect below the transducers [1,2,3,4]. The typical pattern is a windowed waveform that contains almost all the mechanics information of the first defect below the transducers. Meanwhile, for multiple pile defects, the in-hole MPTWD method and its results are utilized to characterize the potential defects one by one from top to toe. Based on the typical pattern, an experienced operator can predict whether there is a defect below the transducers and whether it is a stiffened or weakened defect. However, quantifying the degree of a specific defect is beyond human capacity, even for an experienced expert.
(3) Feature extraction: Two specific techniques for feature extraction are studied in this study. First, the distributed sampling technique through linear interpolation is employed to extract the waveform of the recognized pattern, which is close to the conventional way of visual detection observed by the operators. Second, statistical and signal processing techniques are employed to further reduce the number of features, which makes the features more robust for the machine-learning algorithms. The comparison of the two techniques for feature extraction will be discussed via a case study.
(4) Machine learning and damage characterization: Three classifiers of machine-learning methods are applied to the extracted features and to characterize the pile damage, namely: logistic regression (LR), extreme gradient boosting (XGBoost) and multilayer perceptron (MLP). The pile damage is classified into four categories according to its equivalent cross-sectional acoustic impedance compared with that of the intact part: (a) Class I: |*a_d_*| ≤ 10%, including the intact pile, i.e., *a_d_* = 0; (b) Class II: 10% < |*a_d_*| ≤ 20%; (c) Class III: 20% < |*a_d_*| ≤ 30%; and (d) Class IV: |*a_d_*| > 30%. The defect degree coefficient *a_d_* is defined as

(7) $$a_{d}=\left(1-\frac{Z_{d}}{Z_{p}}\right)\times 100\%=\left(1-\frac{C_{d}r_{d}{}^{2}}{C_{p}r_{p}{}^{2}}\right)\times 100\%,$$

where *Z_d_*, *C_d_*, *r_d_*, *Z_p_*, *C_p_*, *r_p_* are the equivalent acoustic impedance, elastic wave velocity and radius of the first defect below the transducers and the intact segment, respectively.

## 4. Data Acquisition

Wu et al. [16] validated the performance of the MPTWD results derived from the analytical solutions and demonstrated excellent agreement with experimental results. Therefore, the analytical solution is employed herein to generate sufficient samples (up to 10,000) to verify the feasibility of the data-driven modeling framework.

### 4.1. Analytical Solution

**Figure 4:**
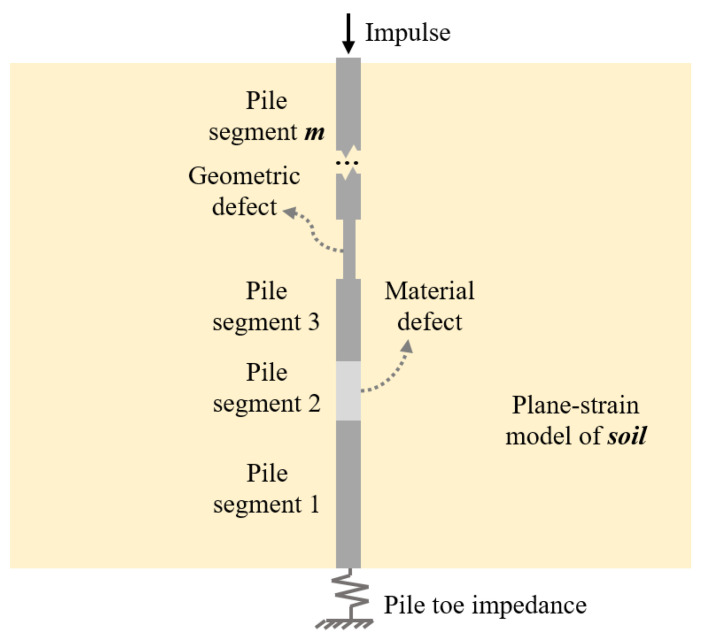
Generalized model for a cylindrical pile with various defects.

Wu et al. [3,14,15] developed an analytical solution for the dynamic response of a cylindrical pile with various defects due to an impulse applied at the pile head as depicted in Figure 4. In this solution, the pile is considered a one-dimensional continuous rod and the surrounding soil is simulated employing the plane-strain model [35].

The pile is divided into *m* segments according to multiple defects and surrounding soil layers, which are denoted as 1, 2,···, *m* from toe to top. The depth variable *z* satisfies the local coordinate system. The general solution to the governing equation of pile segment *i* can be written as

(8a) $$U_{i}=M_{i}\cos\left(\frac{\lambda_{i}}{C_{i}}z\right)+N_{i}\sin\left(\frac{\lambda_{i}}{C_{i}}z\right)$$

(8b) $$\lambda_{i}=\sqrt{\omega^{2}-\frac{2k_{s}}{\rho_{p}r_{i}}},$$

where *U_i_*(*z*,*ω*) = F[*u_i_*(*z*,*t*)] is the Fourier transform of the particle displacement of the pile segment *i*; *M_i_* and *N_i_* are undetermined functions; *ρ_p_* is the mass density of the pile foundation, which is considered to be constant along the pile shaft; *C_i_* and *r_i_* denote elastic wave velocity and the radius of the pile segment *i*, respectively; *ω* is the angular frequency of the harmonic vibration; *k_s_* is the equivalent modulus of pile shaft friction per unit length applied by the surrounding soil, which is a function of the pile radius *r_i_* and shear wave velocity of the surrounding soil *v_si_* and can be determined by the plane-strain model of soil [35].

Neighboring pile segments satisfy the compatibility (displacements) and equilibrium (stress) conditions at interfaces. The impedance transfer mechanism can then be established, where the impedance function *Z_i_* at the top of pile segment *i* is expressed as a function of *Z_i_*_−1_:

(9) $$Z_{i}=\rho_{p}C_{i}\lambda_{i}\cdot \frac{\rho_{p}C_{i}\lambda_{i}\sin\left(\frac{\lambda_{i}}{C_{i}}l_{i}\right)-Z_{i-1}\cos\left(\frac{\lambda_{i}}{C_{i}}l_{i}\right)}{\rho_{p}C_{i}\lambda_{i}\cos\left(\frac{\lambda_{i}}{C_{i}}l_{i}\right)+Z_{i-1}\sin\left(\frac{\lambda_{i}}{C_{i}}l_{i}\right)}.$$

Once the boundary conditions at the pile top and pile toe are given, the undetermined functions can be solved from the pile top to toe. The frequency response function of each pile segment can be obtained by substituting *M_i_* and *N_i_* into Equations (8a) and (8b), and the dynamic response in the time domain can then be solved by applying the inverse Fourier transform (IFT).

### 4.2. Sample Set Generation

In order to obtain a better distribution of the sample set and facilitate the machine learning procedure, the type of defect and its probability of occurrence are artificially controlled. This is similar to the practice of selecting physical samples as needed to build a sample set with better learning ability. The defect types of cast in situ reinforced concrete pile foundations can be roughly divided into two categories: material and geometric defects. The properties of the defect segment can be characterized by the equivalent elastic wave velocity *C_i_* and equivalent pile radius *r_i_* as introduced in the analytical solution.

**Figure 5:**
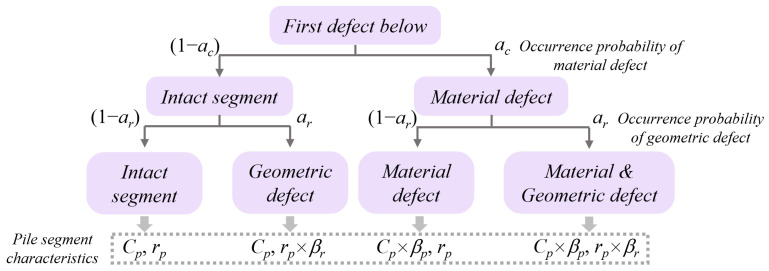
Distribution of sample set on the four different conditions of LPPIT.

The conventional PIT or lateral PIT method is inappropriate for detecting multiple defects, especially when there is a severe defect near the pile top. However, because of eliminating the vibration of the upper part, the in-hole MPTWD method and its result can be employed to detect the first defect one by one by lowering the transducers. From this perspective, it is reasonable to consider only one potential defect between the transducers and the pile toe (or a severe pile defect below). Depicted in Figure 5, in order to manually control the distribution, is the distribution of the sample set on the four different conditions of LPPIT: (1) intact (no defect); (2) material defect only; (3) geometric defect only; and (4) both material and geometric defects. In Figure 5, the two defect-type occurrence probability control parameters *a_c_* and *a_r_* indicate the occurrence probability of the material and geometric defects, respectively; *β_r_* and *β_c_* are the ratios of the radius and elastic wave velocity of the geometric and material defects to that of the intact segment, respectively. For a specific project, the parameters required for the analytical solution (e.g., pile radius, pile length, RC properties, soil properties, test impulse, *β_r_*, *β_c_*, etc.) can be randomly generated within the range given by the geotechnical report and design files.

## 5. Feature Extraction

**Figure 6:**
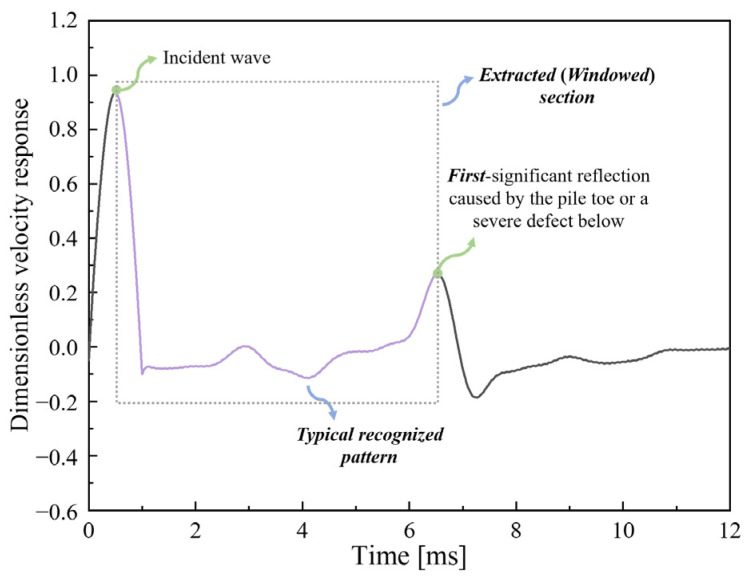
Typical pattern of the in-hole MPTWD result (data from Figure 2c).

In order to identify the reflection caused by the first defect below the transducers, the typical pattern of the in-hole MPTWD result, i.e., the waveform between the incident wave and the first significant reflection (as demonstrated in Figure 6), is utilized to evaluate the mechanics characteristics of the first defect below. Given that the sampling frequency of the MPTWD result is relatively high to maintain all the details, the in-hole MPTWD result, as well as the typical pattern, contain a large amount of raw data. Thus, two specific techniques are introduced herein to further reduce the number of features that are applied to subsequent machine-learning algorithms.

### 5.1. Distributed Sampling

Considering the time period of the typical pattern is inconsistent for various conditions, the distributed sampling method is employed to standardize and reduce the number of features. The raw data are distributed sampled by *N_ds_* points, where the first and last time history velocities are the same as that of the in-hole MPTWD result, while the other values are calculated by linear interpolation. Meanwhile, the amplitude of time history velocity is rescaled by the data length of the typical pattern to account for the effect of the typical pattern time period, i.e.,

(10) $$v_{ds}=\frac{v}{\left(t_{2}-t_{1}\right)/\Delta t+1},$$

where *v_ds_* denotes the rescaled dimensionless velocity considering the typical pattern time period (data length); *t*_1_ and *t*_2_ are the time of the crests of the incident wave and first significant reflection caused by the pile toe or a severe pile defect below, respectively; Δ*t* is the sampling time step of the in-hole MPTWD method, which is set by the user.

**Figure 7:**
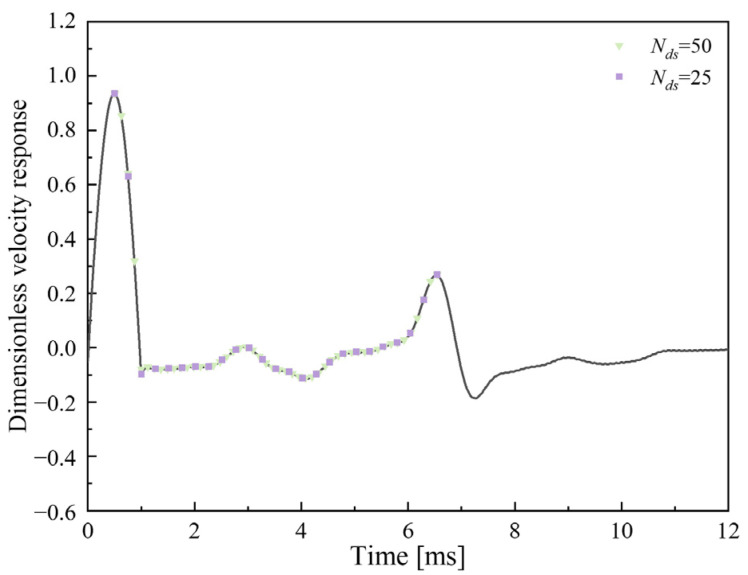
Distributed sampling technique employing different *N_ds_* value (data from Figure 2c).

It should be noted that the sampled (extracted) features are equivalent to the waveform after curve smoothing (filtering). As demonstrated in Figure 7, a larger *N_ds_* value (e.g., *N_ds_* = 50 in this example) will more comprehensively reflect the information of the in-hole MPTWD result while bringing more features, which requires more samples and more complicated learning models. On the contrary, a smaller *N_ds_* value (e.g., *N_ds_* = 25 in this example) will reduce the feature number but the overcompressed (filtered) features will also lose the characteristics of the defects and decrease the accuracy of the machine-learning classifiers. The discussion of the optimal *N_ds_* value is detailed in Section 7.

### 5.2. Statistical and Signal Processing

In order to coordinate the relation between the number of features and the extracted characteristics of the first defect below the transducers, statistical and signal processing techniques are utilized to further optimize the features of the typical pattern.

**Figure 8:**
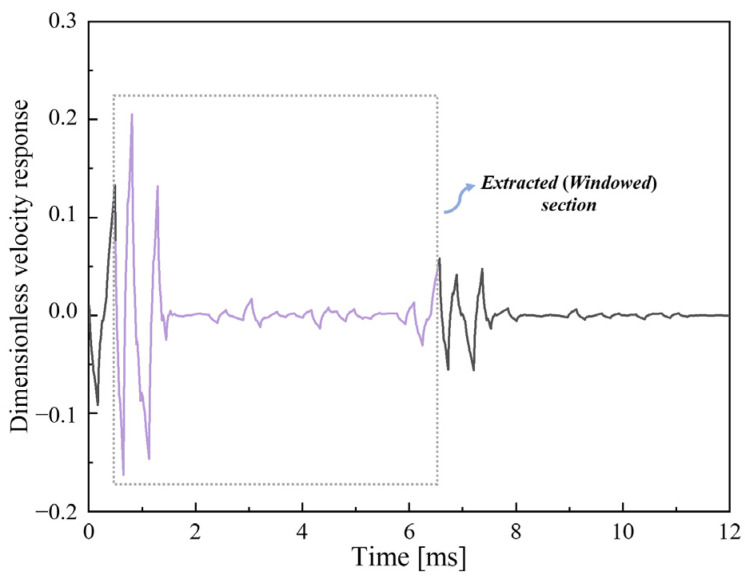
Wavelet transform of the in-hole MPTWD result (five-level decomposition using Symlets 2 (sym2) wavelet).

In addition to the typical pattern, the wavelet transform of the in-hole MPTWD result is employed to identify mutations and fluctuations. Figure 8 displays the five-level decomposition of the in-hole MPTWD result (Figure 2c) using Symlets 2 (sym2) wavelet, and the extracted (windowed) section for the feature extraction, which is the same as that of the typical pattern (Figure 6).

There are a total of 24 features extracted from the typical pattern and detailed wavelet coefficients, which are listed in Table 1.

Considering the incident wave of the in-hole MPTWD result has been standardized already by imposing an idealized semi-sine fictitious excitation, the time (or location) of the crest of the incident wave, i.e., the first greatest peak, almost remains the same for different conditions. Therefore, the time (or location) of the first greatest peak is not selected as the feature. However, the amplitude of the incident wave might be influenced by a defect near the transducers; thus, the amplitude of the first greatest peak is selected as the feature. It is also noted that the spectra are obtained by the complete in-hole MPTWD result and the detailed wavelet coefficients rather than the typical recognized pattern. The upper-frequency limit of our interest in the spectral analysis is 10·*θ_f_*/*π*, which is determined by the duration of the fictitious excitation. The formulations for the features applied to the functions are detailed in the Appendix A.

## 6. Machine Learning and Damage Characterization

The extracted features are applied to multiple machine-learning classifiers to characterize the quantity/degree of the pile damage, where three classifiers are formulated based on logistic regression (LR), extreme gradient boosting (XGBoost) and multilayer perceptron (MLP). The performance of the three classifiers on two types of features is evaluated through a specific case study.

Considering the complexity and uncertainty of the on-site construction, it is not necessary to perform a rigorous regression (i.e., exact value) on the quantity/degree of the pile damage. Furthermore, there is no existing basis for the classification of the RC pile quality, and the construction quality is still evaluated by human experience [23]. It is reasonable to evaluate the potential pile damage from the analytical solution by the equivalent cross-sectional acoustic impedance, and the machine-learning algorithms are then utilized to classify the sample set into four categories (Class I to Class IV). It is noted that the expansion of the pile radius is also considered a defect in this study, which can be readily distinguished by operators or fusion modeling.

The sample set is split into training and testing sets (80%/20%). The *k*-fold cross-validation [36] within the training set is applied to the sample set to tune the hyper-parameters of the machine-learning algorithms. The final model performance is evaluated by the area under the curve (AUC) score, as well as the false positive rate (FPR), false negative rate (FNR) and overall accuracy of classification (ACC) within the test set. The AUC score [37], ranging in value from 0 to 1, is the measure of the ability of a classifier to distinguish between the classes; the higher the AUC score, the better the performance of the model at distinguishing between the positive and negative classes. The FPR, FNR and ACC values derived from the confusion matrix are also used to measure the performance of the classification model and can be calculated by

FPR = False Positive/Negative(11a)

FNR = False Negative/Positive(11b)

ACC = (True Positive + True Negative) / (Negative + Positive).(11c)

## 7. Case Study

A specific case is introduced herein to investigate the feasibility of the data-driven modeling framework and to further improve its performance. The in-hole MPTWD results, derived from the collected time history velocities, which are simulated by the analytical solution, are utilized to detect and characterize the potential damage of floating RC piles embedded in a soft soil profile. The specific value range of the parameters concerned for the generation of the sample set is given as follows.

*C_p_* ∈ [3800 m/s, 4200 m/s], *r_p_* ∈ [0.25 m, 0.50 m], *L* ∈ [8 m, 20 m], *v_s_* ∈ [100 m/s, 200 m/s],

*β_z_* ∈ [0.20, 0.60], *β_r_* ∈ [0.50, 1.50], *β_c_* ∈ [0.50, 1.00], *l_d_* ∈ [0.50 m, 2.00 m], *π/θ_f_* = 1 ms, Δ*t* = 10^−5^ s,

where *L* is the pile length below the middle transducer; *v_s_* denotes the equivalent shear wave velocity of the soil around and beneath the floating pile; *β_z_* is the ratio of the spacing between the middle transducer and the top of the first defect below to *L*; *l_d_* is the length of the first defect below the transducers.

**Figure 9:**
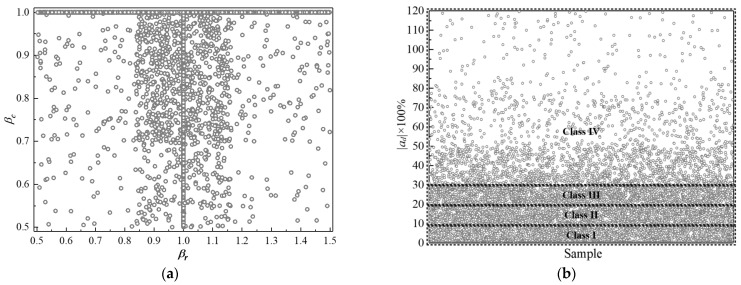
Distribution of 10,000 randomly generated samples on (**a**) defect type; (**b**) defect degree.

Figure 9a,b display the distribution of 10,000 randomly generated samples on the defect type and defect degree, respectively. It can be seen that the sample numbers in four categories (Class I, II, III and IV) are evenly distributed by controlling the occurrence probability of the material and geometric defects (*a_c_*, *a_r_*), as well as the radius and elastic wave velocity ratios (*β_r_*, *β_c_*).

**Figure 10:**
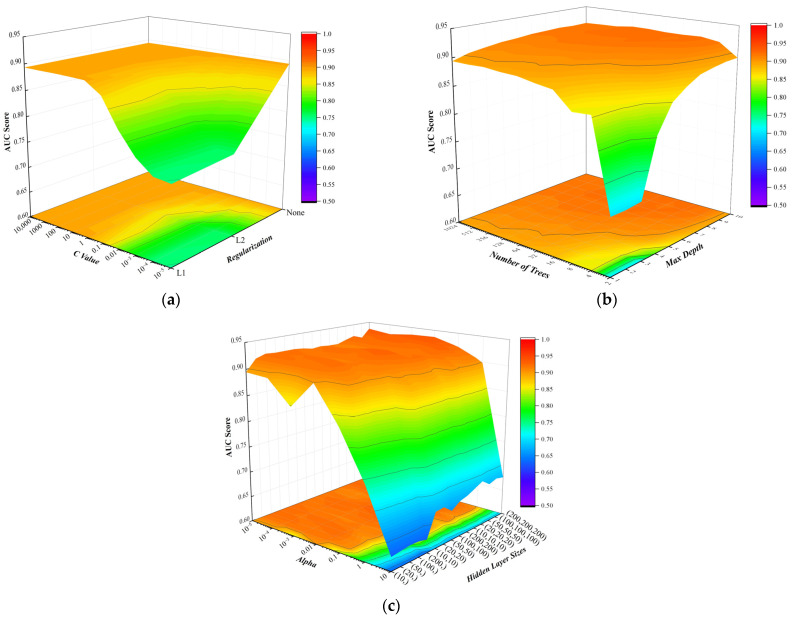
Hyper-parameter optimization of machine-learning algorithms: (**a**) LR; (**b**) XGBoost; and (**c**) MLP.

The randomly generated samples are utilized to tune the hyper-parameters of the machine-learning algorithms by five-fold cross-validation [36,38]. Figure 10a–c demonstrate some specific examples of the hyper-parameter optimization process of the LR, XGBoost and MLP classifiers employing statistical and signal processing techniques, respectively. Figure 10a indicates that the LR classifier performs worse as the regularization becomes stronger, i.e., the C value (inverse of regularization strength) becomes smaller, which means that the overfitting problem is not our concern in this regard. Figure 10b demonstrates that a larger number of trees (more than four) coupled with a deeper max depth (more than five) facilitate the classification. Figure 10c shows that the hidden layer size has an insignificant effect on the performance of the MLP classifier, while the AUC score becomes larger as the alpha value (penalty term) becomes smaller, which is consistent with Figure 10a. The optimal hyper-parameters are determined by searching for the maximum value of the AUC score and are employed for the following study.

**Figure 11:**
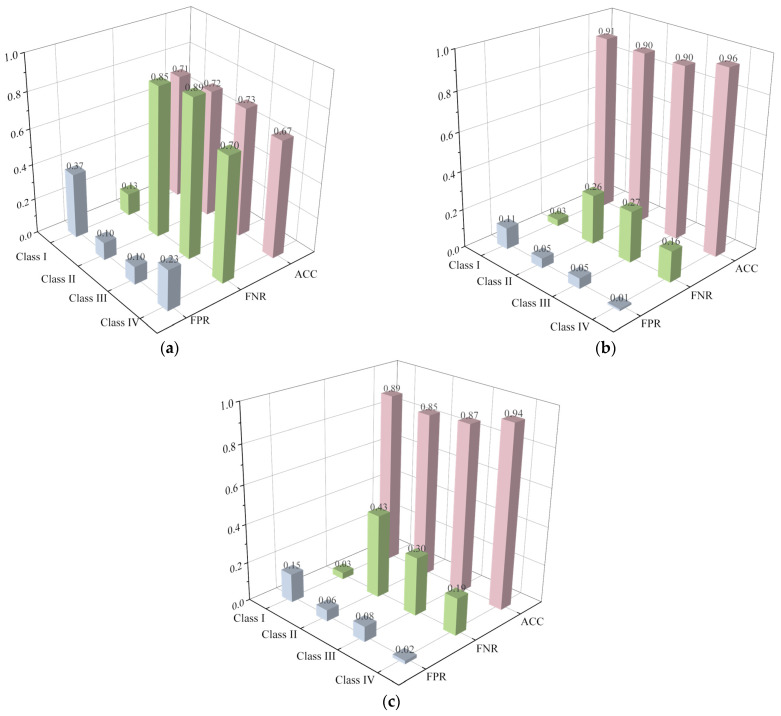
Performance of various machine-learning algorithms: (**a**) LR; (**b**) XGBoost; and (**c**) MLP employing distributed sampling technique (*N_ds_* = 50).

**Figure 12:**
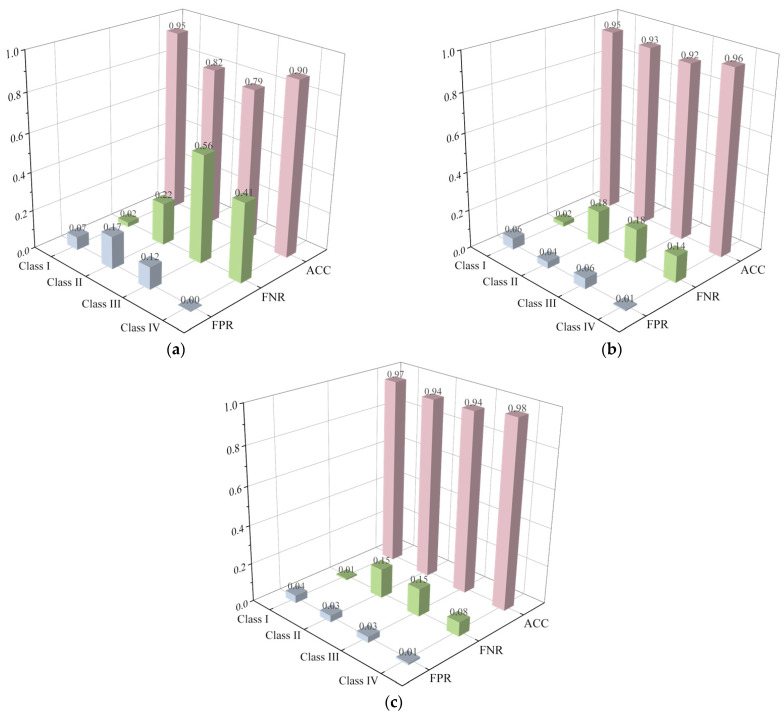
Performance of various machine-learning algorithms: (**a**) LR; (**b**) XGBoost; and (**c**) MLP employing statistical and signal processing technique.

Figure 11 and Figure 12 compare the performance of various classifiers on the randomly generated sample set extracted by distributed sampling and statistical and signal processing techniques, respectively. Considering the sample number is relatively large (10,000 samples), the poor performance of the LR classifier employing the distributed sampling technique (Figure 11a) underscores the complexity and difficulty of the classification problem. Compared with the distributed sampling technique (Figure 11a–c), it can be seen that all the classifiers perform much better when employing the statistical and signal processing technique (Figure 12a–c). Meanwhile, the false positive rates (FPR) of various classifiers on Class I are relatively small (0.07, 0.06 and 0.04) employing the statistical and signal processing technique, which means that few more severely defective segments (Class II, III and IV) are mistaken for almost intact ones (Class I), which is crucial for engineering safety. From this perspective, the extracted 24 features (Table 1) are reasonable and efficient for the machine-learning algorithms.

**Figure 13:**
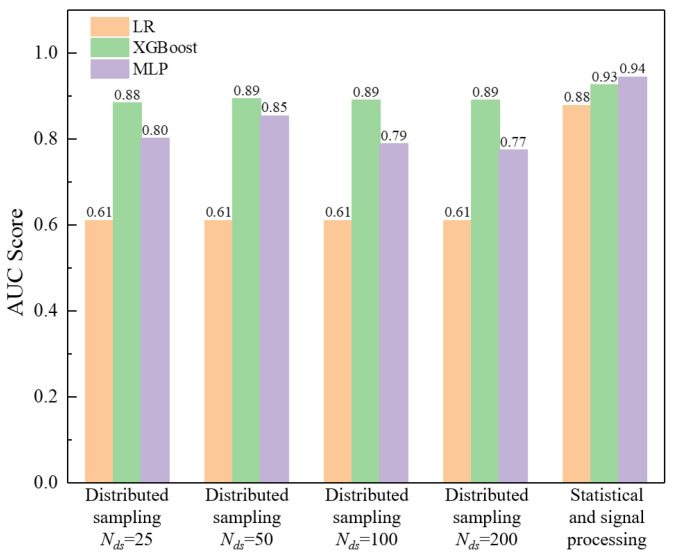
Effect of distributed sampling number *N_ds_* on the performance of various classifiers.

Figure 13 further elucidates the effect of four different distributed sampling numbers *N_ds_* (i.e., 25, 50, 100 and 200) on the performance of various classifiers. It can be seen that the LR classifier is almost unaffected by the *N_ds_* value while the XGBoost and MLP classifers perform best when *N_ds_* is equal to 50. As mentioned before, a larger *N_ds_* value can maintain more details while also reducing the weight of the features, and a smaller *N_ds_* value may filter out features as well; thus, there exists an optimal value (i.e., 50 in this example) for the distributed sampling technique, which limits its application to some extent.

**Figure 14:**
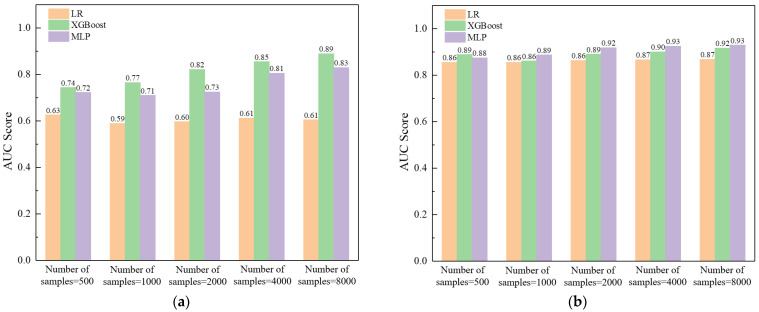
Effect of number of samples on the performance of various classifiers employing: (**a**) distributed sampling and (**b**) statistical and signal processing techniques.

Considering that, in most cases, the number of field test samples is quite limited, the effect of the number of samples on the performance of various classifiers is discussed herein. As expected, the performance of the XGBoost and MLP classifiers improves as the number of samples increases. However, it still needs a large sample size (more than 8000) to obtain satisfactory results employing the distributed sampling technique (Figure 14a). As a comparison, by employing the statistical and signal processing technique (Figure 14b), it is reasonable to establish a sample set of 2000 samples to build a relatively reliable model.

**Figure 15:**
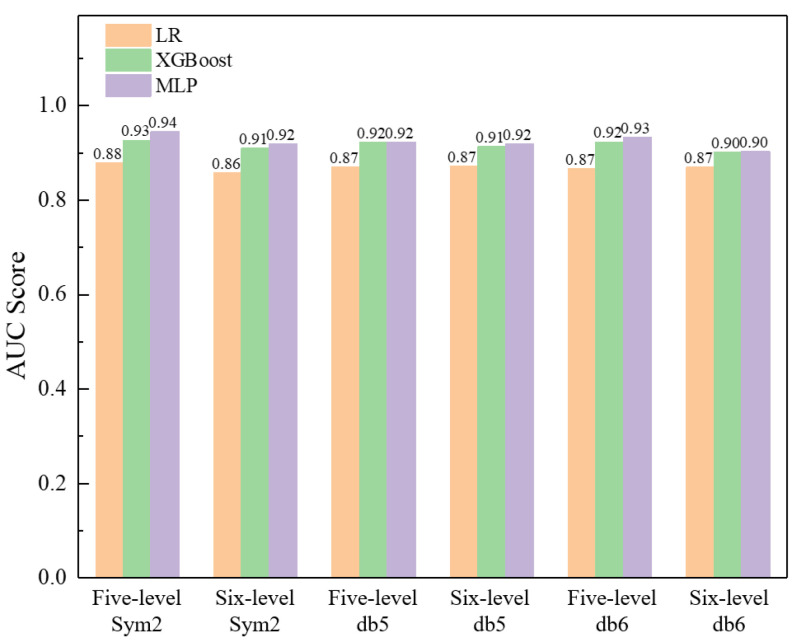
Effect of mother wavelet and decomposition level on the performance of various classifiers employing statistical and signal processing techniques.

Furthermore, Figure 15 investigates the effect of different mother wavelets, e.g., Symlets 2 (sym2), Daubechies 5 (db5), Daubechies 6 (db6), and different decomposition levels (five-level, six-level) on the performance of various classifiers employing the statistical and signal processing technique. Overall, all the detailed wavelet coefficients derived from different mother wavelets, as well as decomposition levels, will facilitate the classification due to their sensitivity to fluctuations and mutations. In this example, it is recommended to employ five-level decomposition using the Symlets 2 (sym2) wavelet to extract the features of the typical pattern.

## 8. Conclusions

(1) The in-hole MPTWD method was developed for the detection and characterization of the potential damage of cast in situ RC piles. It is also suitable for situations involving the lower-part pile integrity test (LPPIT), especially for extremely long piles, piles with multiple defects, a pile head that has not been removed yet or when the reserved sonic logging tubes are partially blocked during the construction period.
(2) Given that the fictitious excitation and time interval of the MPTWD result can be user-defined, the in-hole MPTWD result obtained by the traveling wave decomposition and in-hole MPTWD result reconstruction techniques are ideal for evaluating the lower-part pile integrity and identifying the first defect below the transducers as well as the pile toe.
(3) The in-hole MPTWD result was further applied to establish a data-driven machine-learning framework and to characterize the quantity/degree of the potential damage. The analytical solution to the longitudinal vibration of piles with multiple defects was employed to generate sufficient samples (up to 10,000 in this case study) to verify the feasibility and optimize the performance of the data-driven modeling framework.
(4) Two specific techniques (distributed sampling and statistical and signal processing) were employed to extract features of the typical recognized pattern. A specific case study was conducted to evaluate the performance of the two techniques for feature extraction applied to three machine-learning classifiers. The results show that all three classifiers (LR, XGBoost and MLP) perform much better when employing the statistical and signal processing technique, and the total of 24 extracted features are reasonable and efficient for the machine-learning algorithms.

## Figures and Tables

**Table 1:** List of extracted features.

Functions	Typical Pattern	Detailed Wavelet Coefficients
Types of features (Time domain)	Amplitude of 1st greatest peak Amplitude of 2nd greatest peak Time (or location) of 2nd greatest peak Curve length RMS (root mean square) Skewness Kurtosis	Curve length RMS Skewness Kurtosis Zero crossing rate
Types of features (Frequency domain)	L2 norm Curve length RMS Skewness Kurtosis Zero crossing rate	L2 norm Curve length RMS Skewness Kurtosis Zero crossing rate

Note: 24 features in total.

## Data Availability

No new data were created in this study. Data sharing is not applicable to this article.

## Appendix A

The formulations for the features applied to a discrete signal *x*(*i*) (*i* = 1,2, ···,*n*) are demonstrated in this section.

(A1) $$\mathrm{Curve}\;\mathrm{length}=\sum_{i=2}^{n}\left|x\left(i\right)-x\left(i-1\right)\right|$$

(A2) $$\mathrm{RMS}=\sqrt{\frac{1}{n}\sum_{i=1}^{n}x{\left(i\right)}^{2}}$$

(A3) $$\mathrm{Skewness}=\frac{\frac{1}{n}\sum_{i=1}^{n}{\left[x\left(i\right)-\bar{x\left(i\right)}\right]}^{3}}{{\sqrt{\frac{1}{n-1}\sum_{i=1}^{n}{\left[x\left(i\right)-\bar{x\left(i\right)}\right]}^{2}}}^{3}}$$

(A4) $$\mathrm{Kurtosis}=\frac{\frac{1}{n}\sum_{i=1}^{n}{\left[x\left(i\right)-\bar{x\left(i\right)}\right]}^{4}}{{\left\{\frac{1}{n}\sum_{i=1}^{n}{\left[x\left(i\right)-\bar{x\left(i\right)}\right]}^{2}\right\}}^{2}}$$

(A5) $$L2\;\mathrm{norm}=\sqrt{\sum_{i=1}^{n}x{\left(i\right)}^{2}}$$

where $\bar{x\left(i\right)}$ denotes the mean of the discrete signal *x*(*i*).
